# A high-efficiency *Agrobacterium*-mediated transient expression system in the leaves of *Artemisia annua* L.

**DOI:** 10.1186/s13007-021-00807-5

**Published:** 2021-10-16

**Authors:** Yongpeng Li, Tiantian Chen, Wei Wang, Hang Liu, Xin Yan, Kuanyu Wu-Zhang, Wei Qin, Lihui Xie, Yaojie Zhang, Bowen Peng, Xinghao Yao, Chen Wang, Sadaf-Ilyas Kayani, Xueqing Fu, Ling Li, Kexuan Tang

**Affiliations:** 1 Joint International Research Laboratory of Metabolic and Developmental Sciences, Key Laboratory of Urban Agriculture (South) Ministry of Agriculture, Plant Biotechnology Research Center, Fudan-SJTU-Nottingham Plant Biotechnology R&D Center, School of Agriculture and Biology, Shanghai Jiao Tong University, Shanghai, 200240 China; 2 Center for Plant Biology, School of Life Sciences, Tsinghua University, Beijing, 100084 China

**Keywords:** *Artemisia annua*, Transient transformation, Transcription factor, Promoter activity, Transcription activation

## Abstract

**Background:**

The *Agrobacterium*-mediated transient transformation, which proved effective in diverse plant species, has been widely applied for high-throughput gene function studies due to its simplicity, rapidity, and high efficiency. Despite the efforts have made on *Artemisia annua* transient expression, achieving high-throughput gene functional characterization basing on a fast and easy-manipulated transient transformation system in *A. annua* remains challenging.

**Results:**

The first pair of true leaves of *A. annua* is an ideal candidate for *Agrobacterium* injection. EHA105 was the optimal strain that can be used for the development of the transient expression system. The supplementation of Triton X-100 at a concentration of 0.005% greatly improved the transient expression frequency. According to the histochemical β-Glucuronidase (GUS) staining assay, high transient expression level of the reporter gene (*GUS*) maintained at least a week. Dual-luciferase (Dual-LUC) transient assays showed that the activity of cauliflower mosaic virus 35S (*CaMV35S*) promoter and its derivates varied between *A. annua* and tobacco. In *A. annua*, the *CaMV35*S promoter had comparable activity with double *CaMV35S* promoter, while in tobacco, *CaMV35*S exhibited approximately 50% activity of double *CaMV35S* promoter. Otherwise, despite the *CaMV35S* promoter and double *CaMV35S* promoter from GoldenBraid Kit 2.0 displayed high activity strength in tobacco, they demonstrated a very low activity in transiently expressed *A. annua*. The activity of UBQ10 promoter and endogenous UBQb promoter was investigated as well. Additionally, using our transient expression system, the transactivation of AaGSW1 and AaORA on *AaCYP71AV1* promoter was confirmed. Dual-LUC assays demonstrated that AaHD8 activated the expression of two glandular secreting trichomes-specific lipid transfer protein genes *AaLTP1* and *AaLTP2*, indicating that AaLTP1 and AaLTP2 might serve as downstream components of AaHD8-involved glandular trichome initiation and cuticle formation, as well as artemisinin secretion in *A. annua*.

**Conclusions:**

A simple, rapid, good-reproducibility, high-efficiency and low-cost transient transformation system in *A. annua* was developed. Our method offered a new way for gene functional characterization studies such as gene subcellular localization, promoter activity and transcription activation assays in *A. annua*, avoiding the aberrant phenotypes resulting from gene expression in a heterologous system.

**Supplementary Information:**

The online version contains supplementary material available at 10.1186/s13007-021-00807-5.

## Background

*Agrobacterium tumefaciens* has the ability to integrate transferred DNA (the T-DNA) into its host plant genome, and thus is a commonly used tool for plant genetic transformation [1, 2]. While the stable genetic transformation endows transformed plants with hereditable new characteristics, transient plant transformation mediated by *A. tumefaciens* is widely applied for high-throughput gene function studies due to its simplicity, rapidity and high efficiency [2–4]. In comparison to stable transformation, introduced DNA segments are often expressed at much higher levels, albeit transiently, when introduced into plant cells via transformation [5]. Most of the widely used transient transformation assays involve introduction of *Agrobacterium* into plants using vacuum-aided infiltration technique or a disposable needless syringe [6, 7]. Given the advantages of high efficiency transformation of tobacco, the leaf *agro*-infiltration method was firstly established in *Nicotiana sylvestris* and then adapted for fast gene functional characterization studies [8, 9]. Based on *N. benthamiana* system, researchers have developed varieties of transient expression assays such as protein subcellular localization, protein–protein interaction (Co-immunoprecipitation assay, Co-IP and bimolecular fluorescence complementation assay, BiFC), as well as protein-DNA interaction (dual-luciferase assay), to test gene function and regulation in plant cells in a very short time [10–13]. In addition to tobacco, *agro-*infiltration has proved effective in diverse plant species including *Arabidopsis thaliana* [7, 13, 14], *Vitis vinifera* [15], *Theobroma cacao* [16]*, Gossypium hirsutum* [17]*, Malus domestica* [18] and *Caragana intermedia* [19].

*Artemisia annua*, a widely distributed Chinese medicinal plant belonging to Asteraceae family, is the main and only natural source of artemisinin that is famous for its use as antimalarial drugs [20–22]. Recent years, our group have already identified and characterized a number of transcription factors (TFs) playing significant roles in artemisinin biosynthetic pathway and trichome initiation as well as their transcriptional regulatory networks, using *N. benthamiana* transient expression system [22–28]. However, gene expression in a heterologous system may result in aberrant phenotypes [29] and *N. benthamiana* platforms are not appropriate for studies on species-specific metabolites. Moreover, multigene engineering may require the use of different promoters to avoid the gene silencing caused by the repeated use of the same promoter in the same construct [30]. Transient transformation is an effective approach for fast detection of promoter activity in different plant species and thus provide suitable promoters for plant multigene engineering. For *A. annua* transient transformation, Ma et al. introduced *A. tumefaciens* into leaves cut from 4-week-old aseptic seedlings using vacuum method [31], and our group carried out the transient expression study by transfecting protoplasts that were prepared from 2-week-old *A. annua* mesophyll cells [22]. However, neither method can be considered high throughput as one procedure requires specialized equipment, whereas the other involves complex protoplast isolation. An additional drawback of these two published methodologies is that gene expression can only be monitored for several days before excessive growth of the *Agrobacterium* interferes with the experiment. Given this, a high efficiency and easy manipulated transient expression system in *A. annua* especially *in planta* is needed to be developed.

In the present study, we developed an *Agrobacterium*-mediated leaf transformation method by injecting the first pair of true leaves of 2-week-old *A. annua* seedlings using a 1 mL needless syringe. Specifically, we verified the applicability of our transient expression system on promoter activity detection and transcription activation assays. We compared the activity of various *CaMV35S* promoter and its derivates in tobacco and *A. annua*. Results of our experiments showed that the activity strength of *CaMV35S* promoter and double *CaMV35S* promoter from GoldenBraid Kit 2.0 in *A. annua* was very low. The activity of UBQ10 promoter from *Arabidopsis* and an endogenous polyubiquitin promoter UBQb was also assessed. Moreover, we show that *AaLTP1* and *AaLTP2*, which are involved in artemisinin accumulation and secretion in *A. annua*, are potentially regulated by AaHD8.

## Methods

### Plant materials and growth conditions

*A. annua* L. used for developing transient expression system in this study is a high-artemisinin cultivar, ‘Huhao 1’, which has been subjected to several years selection in Shanghai [32]. Seeds of *A. annua* and *N. benthamiana* were sown in 9 cm pots and grown in a greenhouse as previously described [33].

### Agrobacterium transformation

The *Agrobacterium* strain LBA4404, GV3101 and EHA105 competent cells (Weidi Bio Tech, Shanghai, China) stored at − 80 were thawed on ice and added 5 μL recombined plasmids, then kept in ice for 5 min. Then the mixture was fast frozen in liquid nitrogen for 5 min, followed by an incubation at 37 ℃ for 5 min. After that, the mixture was kept in ice for 5 min and added 300 μL fresh Luria Broth (LB) liquid medium. After a culture in shaker for 1 h at 28 ℃, 200 rpm, 100 μL cells were plated on a LB agar plate containing rifampicin (25 mg/L) and kanamycin (100 mg/L), and then cultured for 2–3 days in dark at 28 ℃.

### Agrobacterium culture and preparation of infiltration

A single positive colony of *Agrobacterium* was inoculated in 1 mL LB liquid medium (25 mg/L rifampicin and 100 mg/L kanamycin) and cultured for 18–24 h in a shaker at 28 ℃, 200 rpm. Then, 500 μL *Agrobacterium* cells were transferred into 10 mL fresh LB liquid medium supplemented with above-mentioned antibiotics and cultured overnight at 28 ℃, 200 rpm. An aliquot of 2 mL *Agrobacterium* cells were pelleted by centrifugation (4000 rpm, 5 min) and re-suspended with 500 μL Murashige and Skoog (MS) medium (3% (w/v) sucrose, pH 5.7). The re-suspended cells were then diluted to OD_600_ = 0.8 with infiltration solution [above-mentioned MS medium, 200 μM acetosyringone (AS), 10 mM MES monohydrate (pH 5.7)]. After a resting of 3 h, the *Agrobacterium* solution was mixed with Triton X-100 at a concentration of 0.005% and ready for infiltration.

### Infiltration of the *A. annua* leaves

The first pair of true leaves of 2-week-old *A. annua* seedlings was transformed by injection of *Agrobacterium* strain cells harboring corresponding plasmids to the abaxial surface using a 1 mL disposable needleless syringe. After injection, the leaf-infiltrated *A. annua* seedlings were dried with paper, covered with a clear plastic lid to keep the humidity, and then moved in dark. After 24-h cultivation, the plants were transferred to greenhouse with a16-h light/8-h dark photoperiod at 23 ± 2 °C for recovery growth. 12 h later, the cover was removed and then the plants were kept under normal growth conditions until sample harvest.

### Fluorescence microscopy

The eGFP was ligated into *pEAQ-HT-DEST1* (GenBank: GQ497235.1) to obtain the *pEAQ-HT-DEST1-eGFP* construct as Stephenson et al., described [34]. Different *Agrobacterium* strains harboring the *pEAQ-HT-DEST1-eGFP* plasmid, were infiltrated into *A. annua* leaves to monitor the transient expression. Pictures were captured at 4 × and 10 × objective with an OLYMPUS BX51 microscope (Tokyo, Japan) at 3 dpi (days post injection). The primers were synthesized by Sangon (Shanghai, China), and all the primers used in this study are listed in Additional file 1: Table S1.

### Luciferase (LUC) assay

For the construction of *pDGB3α1-35S-LUC-Tnos*, GoldenBraid Kit 2.0 [30] was used to assemble DNA elements. CaMV35S promoter from pHB vector was amplified using the primers designed by GoldenBraid 4.0 (https://gbcloning.upv.es) and ligated into the universal acceptor plasmid pUPD2. Next, pUPD2-35S, together with pLuciferase (GB0096) and pTnos (GB0037) (http://www.addgene.org/kits/orzaez-goldenbraid2/#kit-contents) were assembled into pDGB3_alpha1 vector in one reaction. The detailed information for DNA parts assembly method can be found in our previous study [35]. The construct *pDGB3α1-35S-LUC-Tnos* was then respectively introduced into *Agrobacterium* strain LBA4404, GV3101 and EHA105 cells and transiently transformed into *A. annua* leaves to evaluate the transient expression efficiency using LUC assay. Briefly, 1.5 mL Microcentrifuge tubes (NEST, Wuxi, China) containing two steel beads and 0.03 g fresh leaf each sample, accompanied with the steel adaptor, were frozen in liquid nitrogen for several seconds, and immediately placed into the tissuelyser. Then the tissue powder was obtained by fast bead beating for 60 s with 55HZ. LUC activity was measured according to the manufacturer’s instructions (Luciferase Reporter Gene Assay Kit, Yeasen, Shanghai, China). The primers were synthesized by Sangon (Shanghai, China), and all the primers used in this study are listed in Additional file 1: Table S1.

### Western blot analysis

The *A. annua* leaves transiently transformed by *Agrobacterium* strain EHA105 carrying eGFP construct were subjected to protein extraction and Western blot analysis. Two leaves were ground into powder using a tissuelyser as mentioned above. The fine powder was then resuspend using 200 μL Lysis buffer [150 mM Tris–HCl (pH 8.0), 25% Glycerol, 3% PVP, 2% NaCl, 1% Triton X-100, and protease inhibitors including 100 μM Pefabloc (Sigma-Aldrich, USA), 100 μM cocktail (Roche, Switzerland), and 50 μM MG132 (Calbiochem, USA)] and incubated on ice for 1 h. After centrifugation at 14,000 rpm for 10 min, the supernatant was detected using Western blot analysis as previously described [22].

### Histochemical β-glucuronidase (GUS) staining

The *CaMV35S* promoter isolated from pHB vector was cloned into pCAMBIA1391Z vector to drive the expression of *GUS* using ClonExpress^®^II One Step Cloning Kit (Vazyme, Nanjing, China). Then the *Agrobacterium* strain EHA105 harboring *p1391Z*-*35S*-*GUS* plasmid was injected into the leaves of *A. annua* seedlings. The infiltrated leaves were collected after 2–7 days post injection (dpi) and used for histochemical GUS staining assay as previously described [22, 36]. The primers were synthesized by Sangon (Shanghai, China), and all the primers used in this study are listed in Additional file 1: Table S1.

### Dual-LUC transient assay

The *CaMV35S* promoter and double *CaMV35S* promoter from pHB vector as well as *CaMV35S* promoter and double *CaMV35S* promoter from GoldenBraid Kit 2.0, were cloned and ligated into pGreenII0800-LUC vector using ClonExpress^®^II One Step Cloning Kit (Vazyme, Nanjing, China) to drive the expression of *LUC* gene, while the *Renilla* LUC (REN) gene under the control of a *CaMV35S* promoter was used as an internal control [37]. In addition, the promoters of *A. annua polyubiquitin-b* and *Arabidopsis polyubiquitin 10* from GoldenBraid Kit 2.0, were also cloned and ligated into pGreenII0800-LUC vector to investigate their activity in both tobacco and *A. annua* transient expression systems. The promoter sequences can be found in Additional file 1. Two days after injection with *Agrobacterium* strain EHA105 cells harboring the indicated vectors and helper plasmid pSoup 19, the first pair of leaves of *A. annua* seedlings was respectively harvested and ground into fine powder in liquid nitrogen for firefly LUC and REN activities analysis using the Dual-Luciferase^®^ Reporter Assay System (Promega, USA). The relative LUC/REN ratios representing the activity of the promoters were used to evaluate the transient expression system. The primers were synthesized by Sangon (Shanghai, China) and all the primers used in this study are listed in Additional file 1: Table S1.

For the transcriptional activation activity experiments, the full-length coding sequences of *AaGSW1*, *AaORA* and *AaHD8* were inserted into the pHB-YFP (yellow fluorescent protein) vector as effectors, while the promoter sequences of *AaCYP71AVA, AaHD1, AaLTP1 and AaLTP2* were fused to pGreenII0800-LUC vector act as reporters. The detailed information for the constructs can be found in our previous work [23, 25, 26]. For each experiment, four independent plants were, respectively harvested and measured the relative LUC activities. Three independent experiments were performed.

## Results

### The first pair of true leaves of *A. annua* is an ideal candidate for *Agrobacterium* injection

**Fig. 1:**
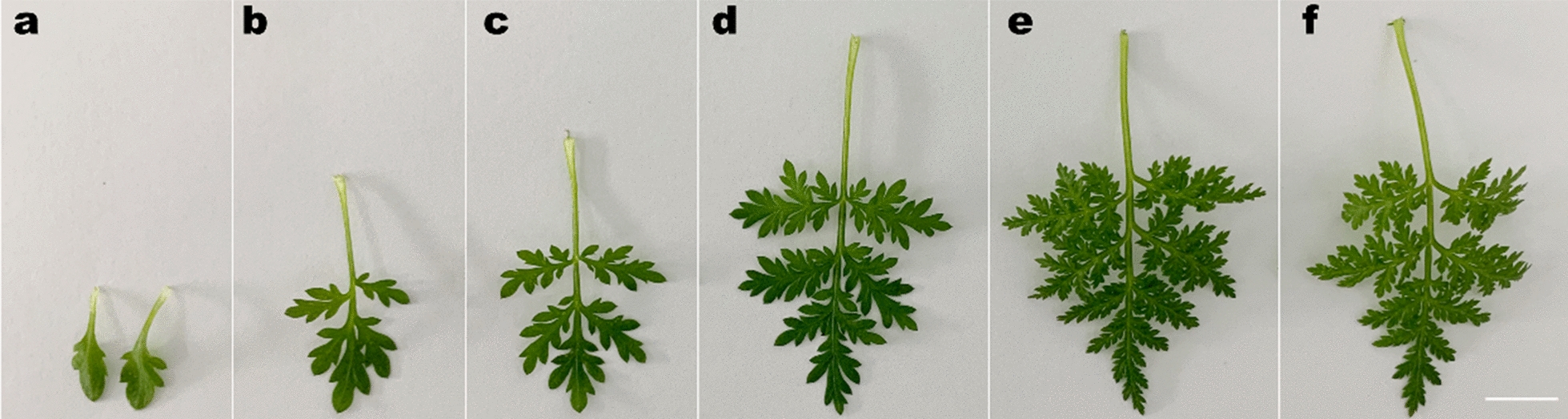
Leaf morphology of 1-month-old *A. annua.* The first pair of true leaves (**a**) and leaf 3–7 (**b–f**). Bar = 1 cm

Normally, the leaves of *A. annua* are odd-pinnately compound with deeply indented margins [38]. Here we found that the first pair of true leaves of *A. annua* exhibits a single and lobed leaf morphology (Fig. 1), which is a good candidate for *Agrobacterium* injection.

### Development of the transient transformation method in *A. annua* leaves

**Fig. 2:**
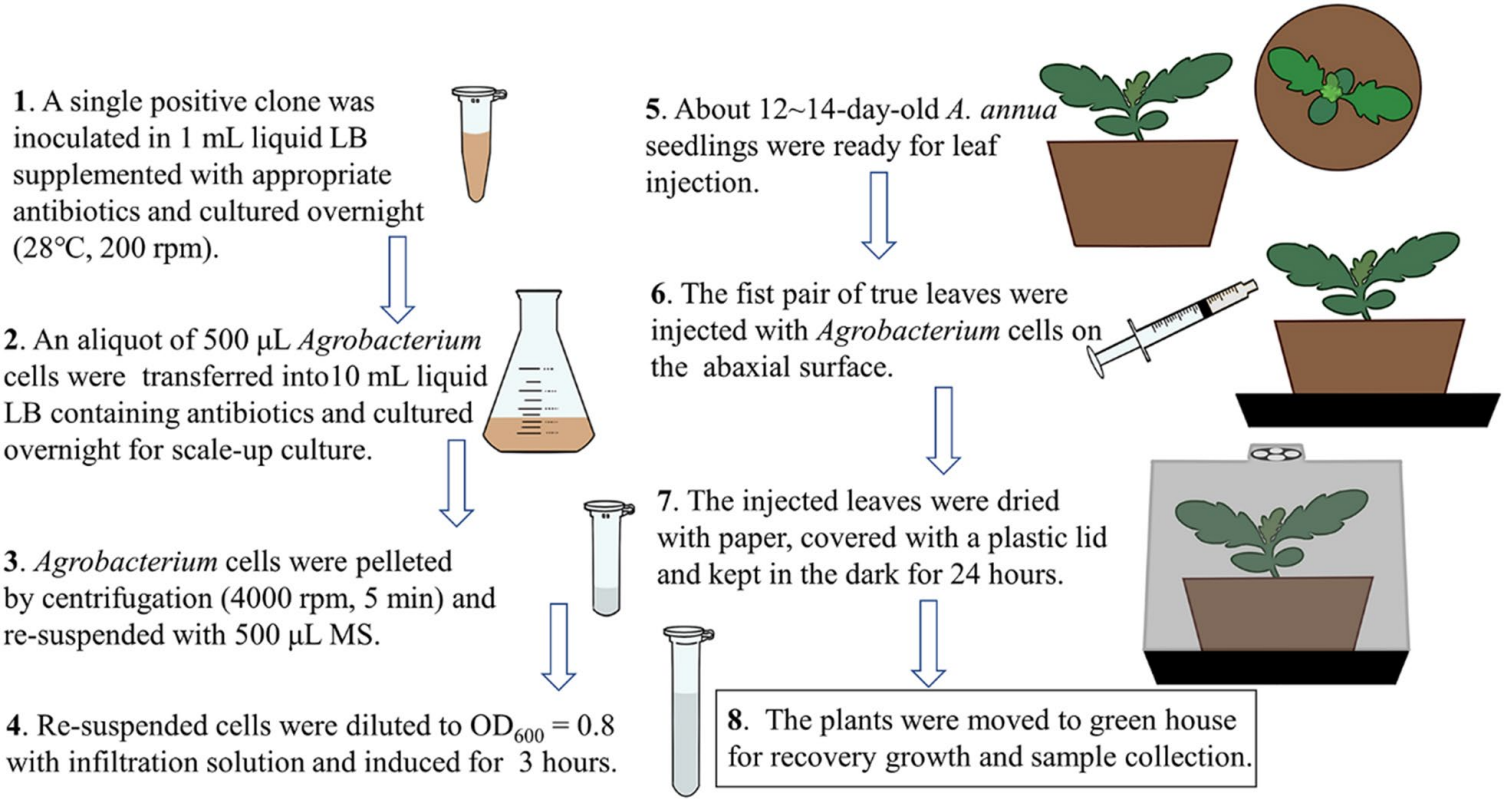
Schematic representation for *Agrobacterium*‑mediated transient transformation of *A. annua*

To determine whether the first pair of true leaves is appropriate for transient expression, three wildly used *Agrobacterium* strains including LBA4404, GV3101 and EHA105 harboring the *pEAQ-HT-DEST1-eGFP* construct were injected into the abaxial surface of 2-week-old *A. annua* leaves. After injection, the plants were moved in dark at 25 ℃ for 24 h. However, leaf wilt symptoms were induced within 1 day post injection (dpi) (Additional file 1: Figure S1). To address this issue, we dried off the injected leaves with paper towel gently, and covered a clear plastic lid to keep the humidity. This greatly improved the recovery growth of infiltrated-*A. annua* seedlings. Therefore, the following transient transformation of *A. annua* was performed according to the workflow depicted in Fig. 2.

**Fig. 3:**
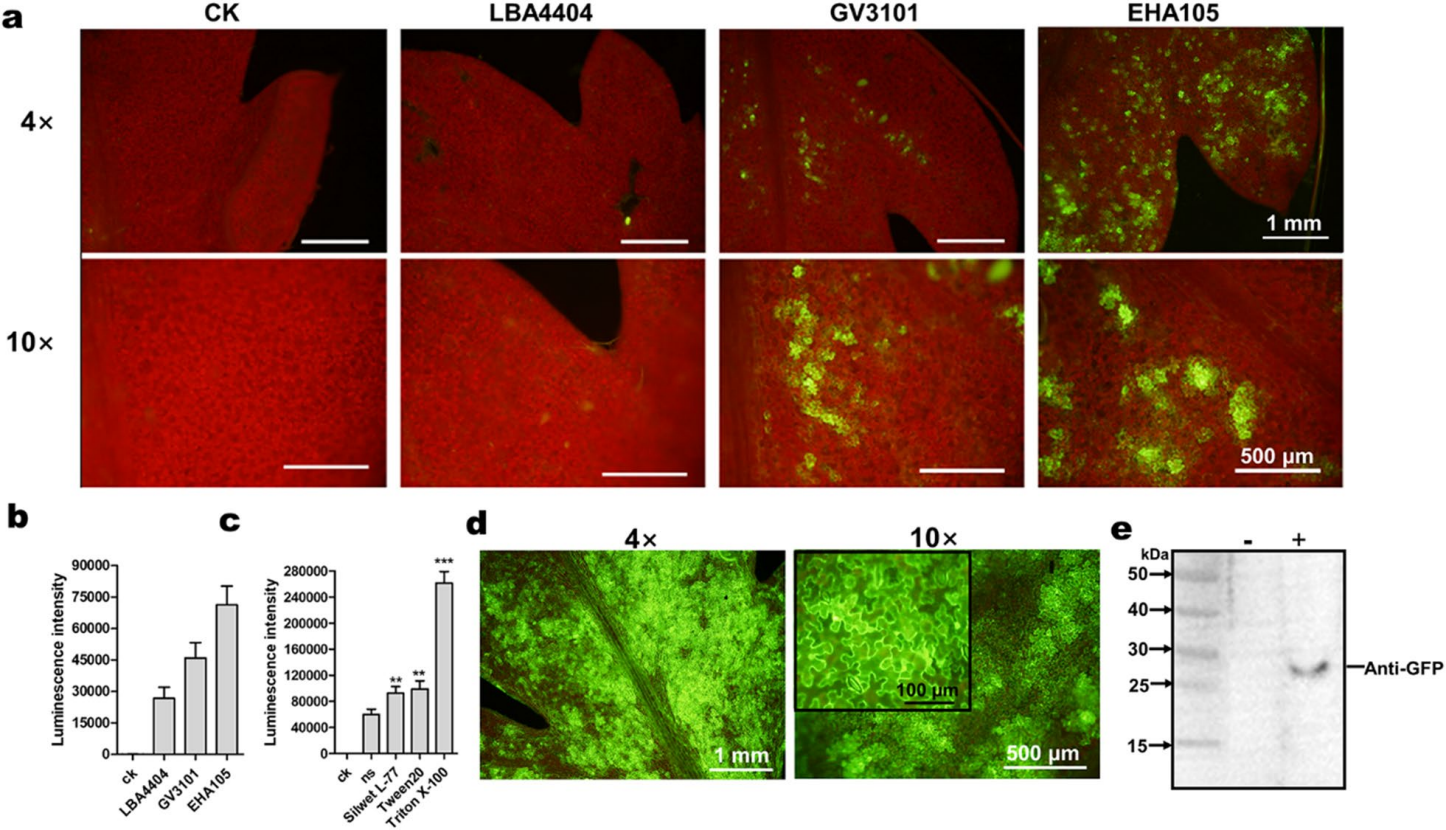
Development of transient expression system in *A. annua* leaves. **a** Leaves infiltrated with *Agrobacterium* strain cells (LBA4404, GV30101, EHA105) carrying *pEAQ-HT-DEST1-eGFP* construct. **b**, **c** Intensity of LUC bioluminescence quantified using GloMax^®^ 20/20 Luminometer (Promega), ck: without leaf-injection, ns: no surfactant. **d** Leaves infiltrated with *Agrobacterium* strain EHA105 cells harboring *pEAQ-HT-DEST1-eGFP* construct using optimized transformation method. **e** Western blot analysis of leaves infiltrated with *Agrobacterium* strain EHA105 cells harboring pEAQ-HT-eGFP construct (+) or no injection (−). Fluorescence microscopy was performed at 3 dpi and images were collected using the 4 × and 10 × objective. Error bars indicate SD (n = 3). Student’s t-test: **P < 0.01

According to the fluorescence microscopy, EHA105-mediated transient transformation of eGFP in *A. annua* showed strongest GFP fluorescent signals, followed by GV3101, while no GFP fluorescent signals were observed in the *A. annua* leaves transiently transformed with LBA4404 (Fig. 3a). Consistently, *A. annua* leaves transiently expressed LUC mediated by EHA105 showed highest LUC activity (Fig. 3b). In addition, the use of surfactants such as Silwet L-77, Tween 20 and Triton X-100 at a concentration of 0.005% drastically improved the transient expression of *LUC* gene, while the addition of Triton X-100 in the infiltration solution resulted in the strongest LUC activity (Fig. 3c). In accordance with the LUC assay, enhanced fluorescence intensity was observed at 3 dpi with the addition of Triton X-100 in the infiltration solution (Fig. 3d). The expression of GFP was further detected using Western blot (Fig. 3e).

**Fig. 4:**
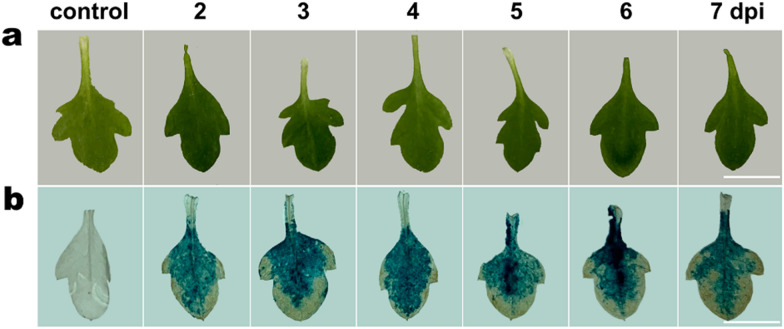
GUS staining analysis of *A. annua* leaves transiently expressing *p1391Z*-*35S*-*GUS* constructs at different days post injection (dpi). **a** Phenotype of *A. annua* leaves at 2–7 dpi and ck (without leaf-injection). **b** GUS staining analysis *A. annua* leaves at 2–7 dpi and control. Bars = 0.5 cm

To further test the transient expression system, leaves injected with *Agrobacterium* EHA105 strain harboring the *p1391Z*-*35S*-*GUS* were, respectively collected at 2, 3, 4, 5, 6 and 7 dpi for GUS staining analysis. GUS staining results showed that, the GUS gene expression can be detected at 2 dpi and maintained a high level until the 7 dpi (Fig. 4).

### Using the *agro-*infiltration method to test promoter activity

**Fig. 5:**
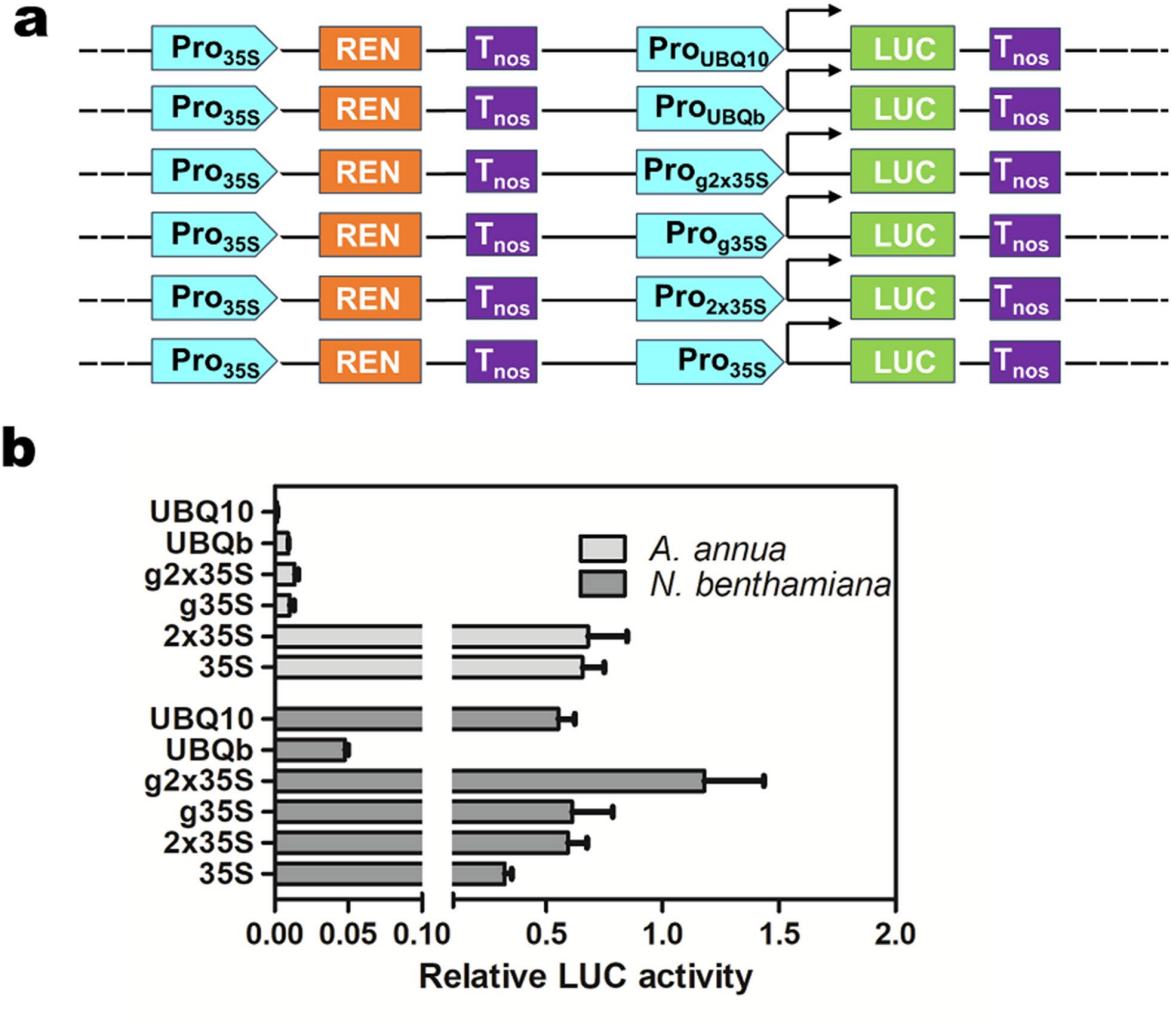
Promoter activity analysis of *CaMV35S* and *polyubiquitin* promoters in *A. annua* and tobacco using Dual-LUC assay. **a** Schematic diagram of the Dual-LUC constructs. **b** Relative LUC activity of *CaMV35S* and *polyubiquitin* promoters in *A. annua* and tobacco. UBQ10, *A. thaliana polyubiquitin 10* (*AtUBQ10*) promoter, UBQb, *A. annua polyubiquitin-b* (*AaUBQb*) promoter, g2 × 35S and g35S represent double and single *CaMV35S* promoter from GoldenBraid Kit 2.0, 35S and 2 × 35S represent the corresponding promoters from pHB vector

To detect the activity of *CaMV35S* promoter and its derivates, as well as *polyubiquitin* promoters in leaves of tobacco and *A. annua*, we measured the firefly LUC and REN activities using Dual-LUC assay (Fig. 5a). As is shown in Fig. 5b, although, double *CaMV35S* promoter was approximately two-fold more active than *CaMV35S* promoter in tobacco, the activity of double *CaMV35S* promoter in *A. annua* was similar to that of *CaMV35S* promoter. Moreover, unlike in tobacco, the *CaMV35S* promoter (g35S) and double *CaMV35S* promoter (g2 × 35S) from GoldenBraid Kit 2.0 displayed strong activity, a very low activity was observed in *A. annua.* In addition to *CaMV35S* promoters, *Arabidopsis ubiquitin* promoter (UBQ10) was found suitable for plant transformation studies [39, 40]. Therefore, the characterization of endogenous *ubiquitin* promoter would be beneficial for future studies of *A. annua.* Based on the RNA-seq data [41], we observed that the expression of *Aannua02727S332000,* which is homologous to *Arabidopsis UBQ10*, is lower than *Aannua01692S241190* (*polyubiquitin-b*) in all the tested organs/tissues of *A. annua* (Additional file 1: Figure S2). Then, the promoter of *polyubiquitin-b* (UBQb), together with *Arabidopsis* UBQ10 from GoldenBraid Kit 2.0 were cloned for activity analysis. In tobacco, UBQ10 exhibited higher promoter activity than UBQb, and is comparable with g35S, while in *A. annua* the activity of UBQ10 is lower than UBQb (Fig. 5b).

### Using the *agro*-infiltration method to test transcriptional activation activity

**Fig. 6:**
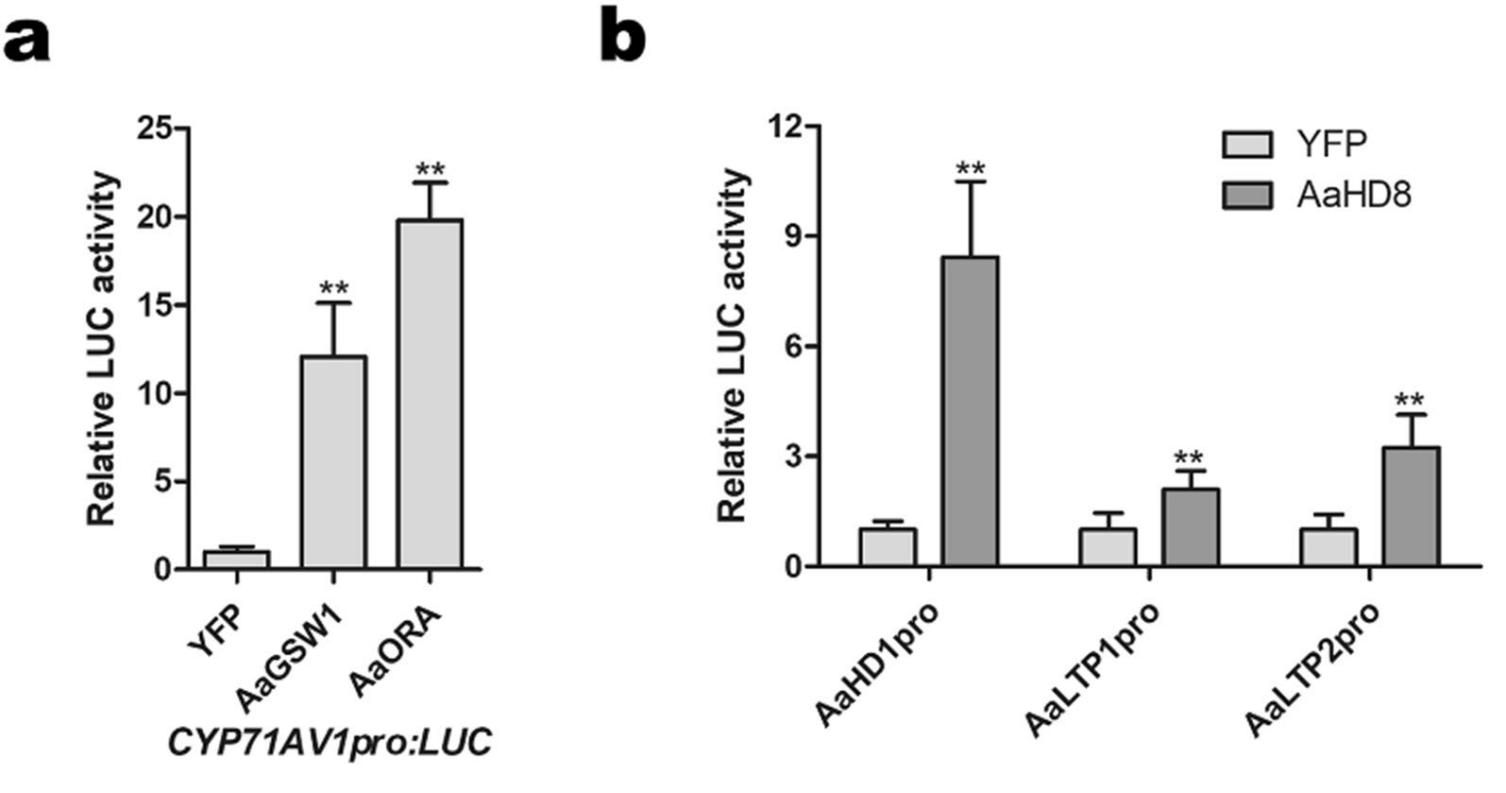
Dual-LUC assay in *A. annua* cells. **a** Activation of *CYP71AV1* gene promoter by AaGSW1 and AaORA. **b** Activation of AaHD1, AaLTP1 and AaLTP2 gene promoters by AaHD8. The pHB-YFP was used as a negative control. The promoters of *AaCYP71AVA*, *AaHD1*, *AaLTP1* and *AaLTP2* were fused to drive the expression of Firefly luciferase (LUC) gene while the Renilla luciferase (REN) gene under the control of a CaMV35S promoter was used as an internal control, and the LUC/REN ratio of YFP was set as 1. Error bars indicate SD (n = 3). Student’s t-test: **, P < 0.01

We have previously showed AaGSW1 and AaORA regulate the artemisinin biosynthesis by directly binding to the promoter of *CYP71AV1* and activating its expression [23, 26]. To test the applicability of our transient expression system, Dual-LUC assays were conducted to confirm the interaction of AaGSW1 and AaORA and *AaCYP71AV1* promoter. As expected, both AaGSW1 and AaORA significantly activated the expression of *AaCYP71AV1* promoter (Fig. 6a).

Next, we employed this transient transformation method to investigate a new transcriptional regulation relationship in artemisinin biosynthesis and accumulation pathways in *A. annua*. AaHD8, a homeodomain‐leucine zipper (HD‐ZIP) IV transcription factor, has been previously reported to boost the artemisinin production by activating the expression of *AaHD1*, which played important roles on promoting glandular trichome initiation [25]. The regulatory network of AaHD8 enhancing the artemisinin accumulation by increasing trichome density has been sufficiently elucidated. However, the underlying mechanism involved in artemisinin secretion modulated by AaHD8 remains unclear. According to the comparison between *AaHD8*‐silenced lines and WT plants transcriptome data, we found two lipid transfer protein genes *AaLTP1* and *AaLTP2*, which are specifically expressed in glandular secreting trichome (GST), were significantly downregulated by 114 and 86-fold, respectively [25]. Furthermore, according to the RNA-seq data for seed, root, leaf, bud and trichome [41], *AaLTP1* and *AaLTP2* showed similar expression pattern with *AaHD8,* as well as the four structural artemisinin biosynthesis genes *ADS*, *CYP71AV1*, *DBR2* and *ALDH1* in the examined organs/tissues (Additional file 1: Figure S3). Meanwhile, in the *AaLTP1-*overexpressed lines, pronounced enhancement of artemisinin production was observed (unpublished data). Here, we carried out Dual-LUC assays in *A. annua* leaves to assess the capacity of AaHD8 to transactivate the *AaLTP* promoters. As we can see in Fig. 6b, AaHD8 significantly activated the expression of *AaLTP1* and *AaLTP2*, as well as *AaHD1* promoters in *A. annua* leaves.

## Discussion

Transgenic plants are widely used to produce valuable metabolites or functional proteins [42, 43]. In addition to the application on production, transgenic technology is a very important approach to introduce useful traits into plants [44–46] as well as characterize the function and regulation of novel genes [47, 48]. However, for many plant species, stable transformation is time-consuming and unsuitable for large-scale analyses [29]. *Agrobacterium*-mediated transient transformation is capable of expressing the target genes transiently with a high level, and hence is facile and versatile for the high-throughput gene functional characterization in plants, such as promoter activity, transcriptional regulation, protein subcellular localization and protein–protein interaction [19]. Researchers have well established the leaf *agro*-infiltration transient expression system in *N. benthamiana*, which is a commonly used platform for transient gene expression analysis [49]. Despite the advantages offered by tobacco transient expression system, a homologous gene transfer system is significantly needed for each plant species to avoid the undesirable affects caused by heterologous gene expression [50, 51].

*A. annua* is generally recognized as a safe plant source for the extraction and purification of artemisinin, which has important roles in combating malaria [52, 53]. Moreover, the recently published genome sequence of *A. annua* [41] was believed to accelerate the research on gene function and promoter characterization. Previous studies have proved that promoter activity varied in different plant species. For instance, the most frequently used promoter *CaMV35S* could constitutively drive high level of gene expression in dicots, however less effective in monocots, such as rice and corn [54]. Besides, GUS activity in transgenic chrysanthemums using the *CaMV35S* promoter was low [55]. Similar results were also observed in chickpea [56]. Notably, along with the rapid development of DNA assembly technologies, multigene transformation which has the ability to introduce more complex and ambitious phenotypes into transgenic plants, is becoming more and more frequently-used in plant biotechnology [57]. However*,* repetitious promoter use in one construct may cause homology dependent gene silencing [58]. Therefore, it is necessary to seek more available promoters with desired activity strength.

Detecting the promoter activity using transient expression system has been well studied in many plant species [11, 14, 37, 59–61]. For *A. annua*, due to its odd-pinnately compound leaves with deeply indented margins, it is not appropriate for *Agrobacterium* injection (Fig. 1). Ma et al. performed the transient transformation by introducing *A. tumefaciens* into the leaves cut from 4-week-old aseptic seedlings using a vacuum pump [31], whereas our previous transient expression assays relied on protoplasts which were prepared from 2-week-old *A. annua* mesophyll cells as recipient cells [22]. Here, we developed a time-saving and easy-manipulated transient transformation method without the employ of additional equipment or isolation of mesophyll protoplasts. We found that the first pair of true leaves of *A. annua* was the most suitable material for *Agrobacterium* injection, which could also minimize the time required to conduct transient transformation assay. However, leaf necrosis was observed when using conventional transient transformation method (Additional file 1: Fig. S1). To avoid the leaf necrosis resulted from *Agrobacterium* injection, we used a paper towel to soak up the residual *Agrobacterium* fluid and kept the seedlings in a relative high humidity (Fig. 2), which was very helpful for the recovery growth of injected *A. annua* leaves. Among the three *Agrobacterium* strains chosen for transient transformation, EHA105 was the optimal strain that can be used for the development of the transient expression system (Fig. 3a, b). Nevertheless, GFP fluorescence was merely observed in occasional *A. annua* cells (Fig. 3a). Given that the presence of surfactants greatly improved transient transformation efficiency, we explored the use of Silwet L-77, Tween 20 and Triton X-100 which were helpful for bacteria to enter the intercellular space [13, 17, 62]. LUC assay showed that all the three surfactants were able to improve the efficiency of transient transformation in *A. annua* (Fig. 3c)*.* Supplementation of Triton X-100 elevated the transient expression most markedly, which is similar to the results observed in transiently transformed *Arabidopsis* [62]. GUS staining results showed the reporter gene (*GUS*) continuously expressed and maintained its expression at least a week (Fig. 4). Moreover, our method can be used for promoter activity detection and transcriptional activation assays.

*CaMV35S* promoter and its derivates are constitutively expressed promoters widely used in plant biotechnology [63]. Intriguingly, we found *CaMV35S* promoter was effective as double *CaMV35S* promoter *A. annua*, whereas the activity of *CaMV35S* promoter was about 50% of double *CaMV35S* promoter in tobacco. Additionally, the *CaMV35S* promoter (g35S) and double *CaMV35S* promoter (g2 × 35S) from the GoldenBraid Kit 2.0 could drive higher level of heterologous gene expression in tobacco plants, than that of *CaMV35S* promoter and double *CaMV35S* promoter from pHB vector. However, the luciferase gene expression under the control of g35S and g2 × 35S promoters in *A. annua* was very low (Fig. 5). Similarly, despite the strong activity of *Arabidopsis* UBQ10 promoter exhibited in tobacco, an extremely low activity was observed in *A. annua.* Furthermore, in *A. annua* transient expression system, the endogenous *polyubiquitin* promoter UBQb showed higher activity than *Arabidopsis* UBQ10 promoter*.* These results indicated that promoter activity was different in various genetic background. Thereby, the activity of promoters in a plant species needs to be investigated prior to being applied for further study.

We also report here that this transient transformation method can be used to test transcriptional activation activity. As is shown in Fig. 6a, AaGSW1 and AaORA positively regulate the expression of *AaCYP71AV1*, which is in accordance with our previous studies. Overexpression of *AaLTP3* and *AaLTP4* have proved to result in the increase of sesquiterpene lactones production including arteannuin B, artemisinin, dihydroartemisinic acid and artemisinic acid [64]. Nevertheless, *AaLTP3* is specifically expressed in non-GSTs, while *AaLTP4* is expressed in both non-GSTs and GSTs, while artemisinin is mainly synthesized and accumulated in GSTs. Recently, two GSTs-specific *AaLTPs AaLTP1* and *AaLTP2* were functionally characterized (unpublished data). As expected, *AaLTP1*-overexpressed lines exhibited higher artemisinin accumulation as compared with the WT plants. Since overexpression of AaHD8 increased the expression of *AaLTP1* and *AaLTP2* drastically, we speculated that AaHD8 appeared to be transcription activator of *AaLTP1* and *AaLTP2* and therefore regulated glandular trichome initiation and sesquiterpene lactone secretion, resulting in elevated artemisinin accumulation. Consistent with this interpretation, based on the Dual-LUC assay results, AaHD8 significantly boosted the expression of *AaLTP1* and *AaLTP2* promoters (Fig. 6b), indicating that AaHD8 was a positive regulator of *AaLTP1* and *AaLTP2*. However, clarification of the regulatory relationship among them still needs further investigation. It should be mentioned that the recent release of genome information about *A. annua* by our laboratory provides a powerful platform for *A. annua* genetics study and breeding, and we strongly believe that this method will prove highly useful for future research performed on *A. annua*.

## Conclusion

In summary, we developed a simple, rapid, high-efficiency and easy-manipulated transient expression system in *A. annua*. Our method can be used for gene functional characterization studies such as promoter activity detection and transcriptional activation assays in *A. annua*, avoiding the aberrant results caused by gene expression in a heterologous system. Moreover, our transient expression system provides a new way for fast characterization of putative promoters with desirable activity in *A. annua*, which is necessary to overcome gene silencing resulting from the use of multiple copies of the same promoter in multigene engineering.

## Supplementary Information

**Additional file 1: Figure S1** Necrosis was observed in leaves infiltrated with *Agrobacterium* cells harboring *pEAQ-HT-DEST1-eGFP* construct at 24 hours post injection. **Figure S2** Heatmap of the expression levels of *A. annua* polyubiquitin genes in different organs/tissues. **Figure S3** Heatmap of the expression levels of *A. annua* *LTP2*, *LTP1* and *HD8,* as well as four structural artemisinin biosynthesis genes in different organs/tissues. **Table S1** Primers used in this article.

## Data Availability

All data generated or analyzed during this study are included in this published article.
