# Supplementary material for: A high-efficiency Agrobacterium-mediated transient expression system in the leaves of Artemisia annua L
Source: Plant Methods. 2021 Oct 16;17:106. doi: 10.1186/s13007-021-00807-5 (PMC8520255; doi:10.1186/s13007-021-00807-5)
Supplement: Supplementary file 1 — Additional file 1: Figure S1 Necrosis was observed in leaves infiltrated with Agrobacterium cells harboring pEAQ-HT-DEST1-eGFP construct at 24 hours post injection. Figure S2 Heatmap of the expression levels of A. annua polyubiquitin genes in different organs/tissues. Figure S3 Heatmap of the expression levels of A. annua LTP2, LTP1 and HD8, as well as four structural artemisinin biosynthesis genes in different organs/tissues. Table S1 Primers used in this article. [file 13007_2021_807_MOESM1_ESM.docx]

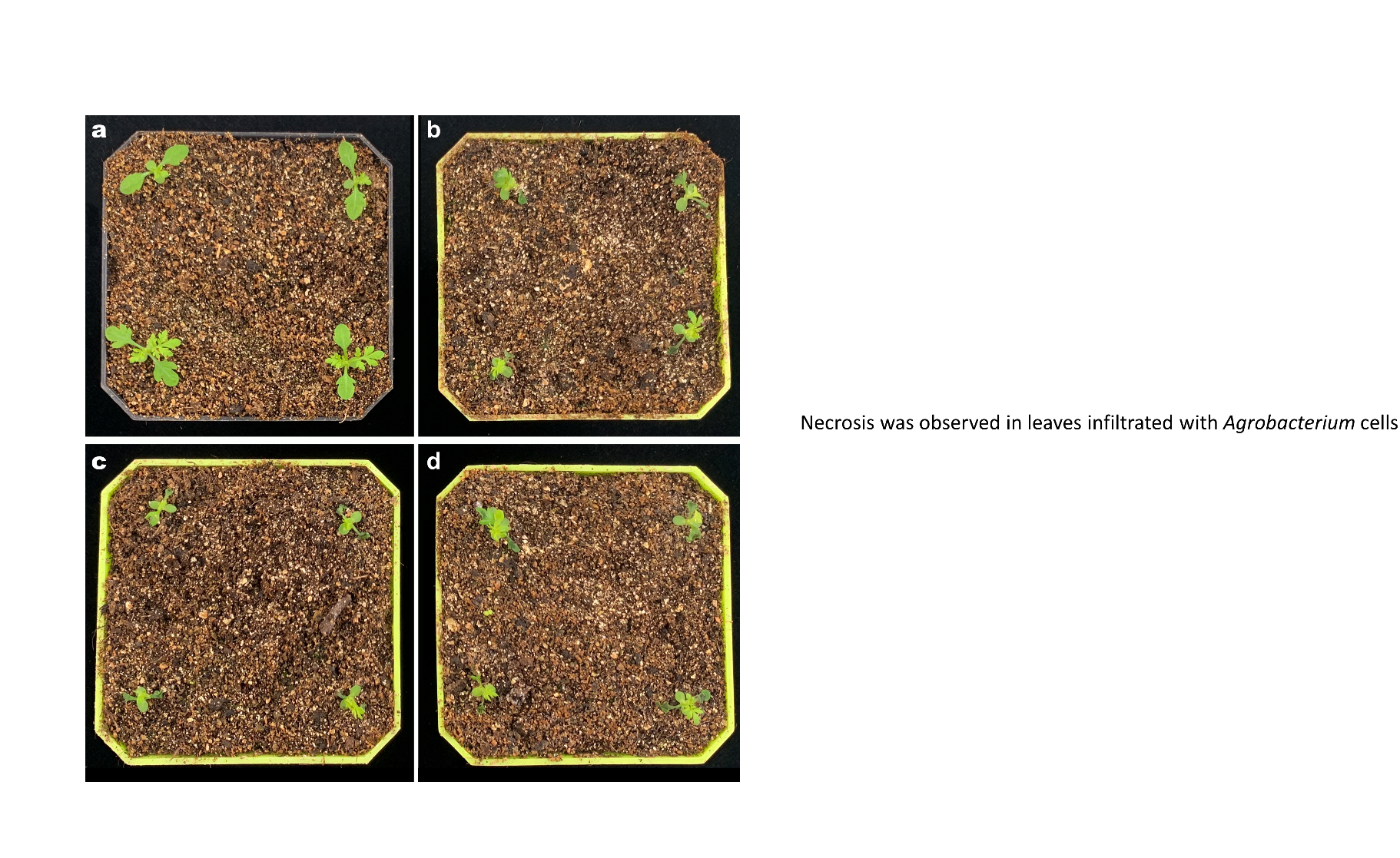


**Figure S1** Necrosis was observed in leaves infiltrated with *Agrobacterium* cells harboring *pEAQ-HT-DEST1-eGFP* construct at 24 hours post injection. **a** Control (seedlings without leaf-injection), **b** Seedlings injected with *Agrobacterium* strain LBAEE04, **c** Seedlings injected with *Agrobacterium* strain gv3101, **d** Seedlings injected with *Agrobacterium* strain EHA105.


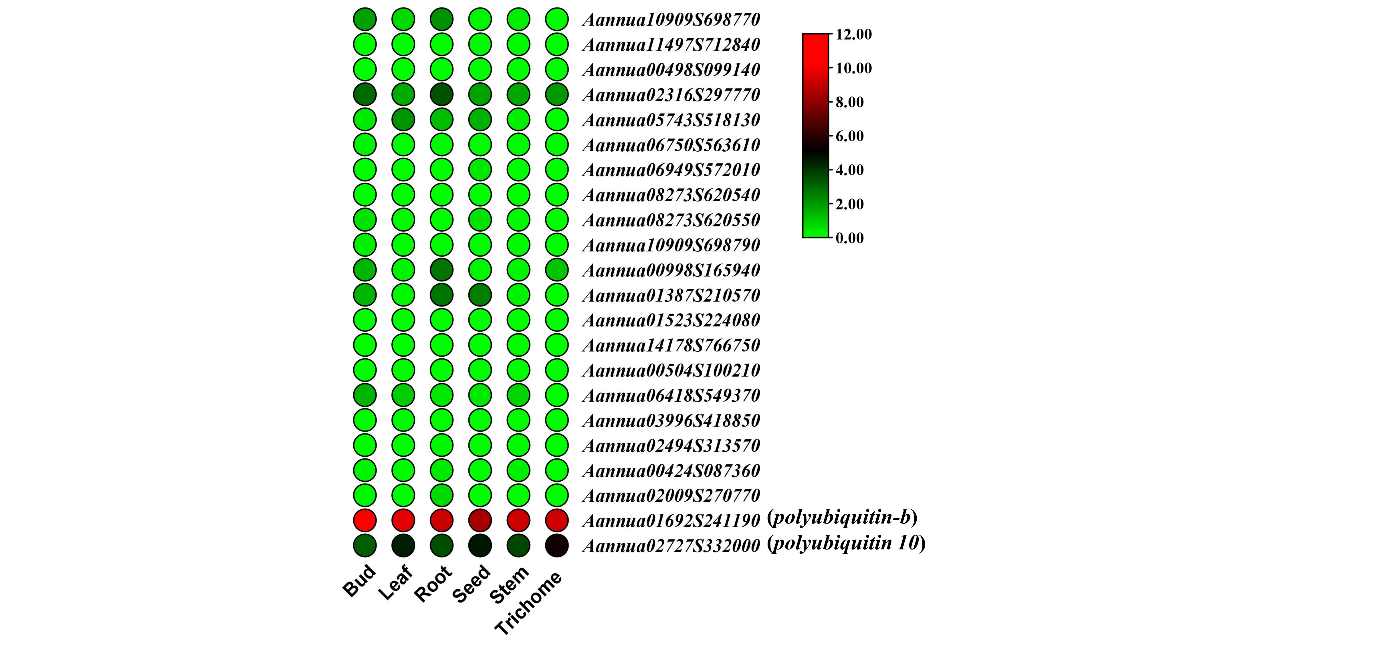


**Figure S2** Heatmap of the expression levels of *A. annua* polyubiquitin genes in different organs/tissues. The color scale on the right represents the RPKM (reads per kilobase per million mapped reads) values. Data is processed by log2. The heatmap was constructed using TBtools.


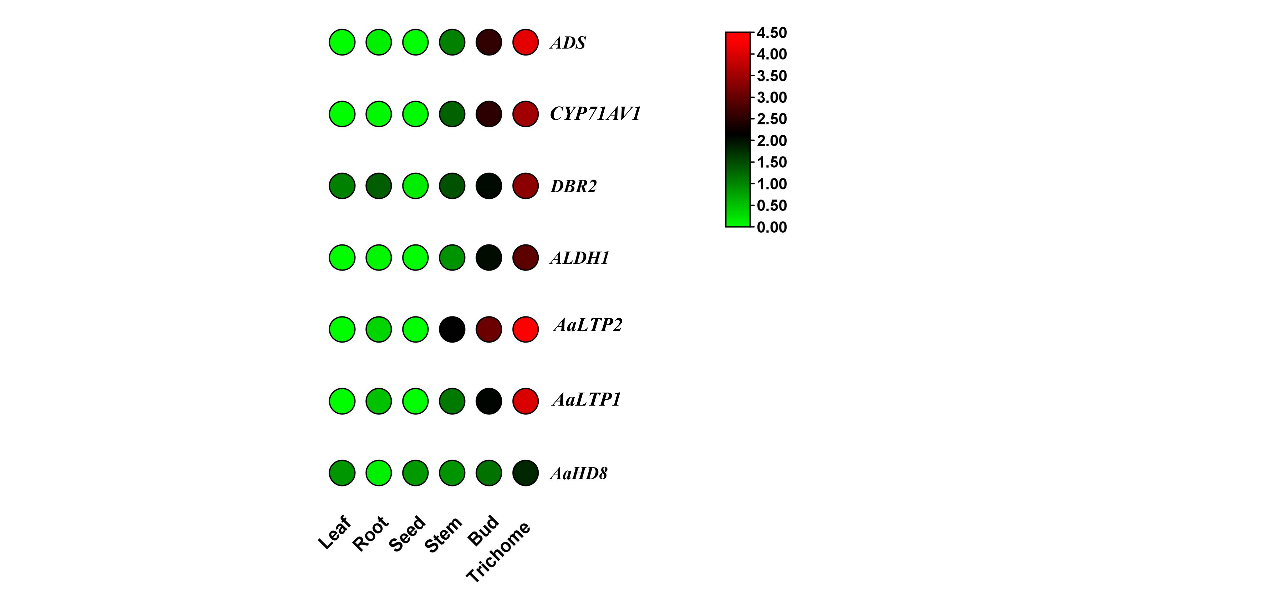


**Figure S3** Heatmap of the expression levels of *A. annua* *LTP2*, *LTP1* and *HD8,* as well as four structural artemisinin biosynthesis genes in different organs/tissues. The color scale on the right represents the RPKM (reads per kilobase per million mapped reads) values. Data is processed by log10. The heatmap was constructed using TBtools.

**Table S1 Primers used in this article**

| **Primer** | **Sequence (5’-3’)** | **Purpose** |
| --- | --- | --- |
| eGFP-F | GGGGACAAGTTTGTACAAAAAAGCAGGCTTAATGGTGAGCAAGGGCGAGGA | pDONR-eGFP construction |
| eGFP-R | GGGGACCACTTTGTACAAGAAAGCTGGGTATCACGAAGCCTTGTACAGCT | pDONR-eGFP construction |
| Pupd2-35S-F | GCGCCGTCTCGCTCGGGAGCATGGAGTCAAAGATTCAAATAGA | Pupd2-35S construction |
| Pupd2-35S-R | GCGCCGTCTCGCTCACATTCGTGTTCTCTCCAAATGAAATG | Pupd2-35S construction |
| 1391-35S-F | CAGGTCGACGGATCCCATGGAGTCAAAGATTC | p1391Z-35S construction |
| 1391-35S-R | TCAGATCTACCATGGCGTGTTCTCTCCAAATG | p1391Z-35S construction |
| 0800-35S-F | CAGCCCGGGGGATCCCATGGAGTCAAAGATTC | pGreenII0800-35S construction |
| 0800-35S-R | TGGCGTCTTCCATGGCGTGTTCTCTCCAAATG | pGreenII0800-35S construction |
| 0800-2×35S-F | CAGCCCGGGGGATCCGAATTCGCCCGGGGATC | pGreenII0800-2×35S construction |
| 0800-2×35S-R | TGGCGTCTTCCATGGAGAGAGAGACTGGTG | pGreenII0800-2×35S construction |
| 0800-g35S-F | CAGCCCGGGGGATCCGGAGACTAGAGCCAAG | pGreenII0800-g35S construction |
| 0800-g35S-R | TGGCGTCTTCCATGGTCGACTAGAATAGTAAAT | pGreenII0800-g35S construction |
| 0800-g2×35S-F | CAGCCCGGGGGATCCGGAGACTAGAGCCAGCTG | pGreenII0800-g2×35S construction |
| 0800-g2×35S-R | TGGCGTCTTCCATGGTCGACTAGAATAGTAAAT | pGreenII0800-g2×35S construction |
| 0800-UBQb-F | CAGCCCGGGGGATCCGTTTGTTTTGGCTTATTTAC | pGreenII0800-UBQb construction |
| 0800-UBQb-R | TGGCGTCTTCCATGGCTGCACAAAATAAAAAATC | pGreenII0800-UBQb construction |
| 0800-UBQ10-F | CAGCCCGGGGGATCCGGAGGTCGACGAGTCAGT | pGreenII0800-UBQ10 construction |
| 0800-UBQ10-R | TGGCGTCTTCCATGGTCTGTTAATCAGAAAAACTC | pGreenII0800-UBQ10 construction |
| 0800-LTP1-F | CAGCCCGGGGGATCCTTACACGTTCTTACTTTAATTT | pGreenII0800-Pro_LTP1_ construction |
| 0800-LTP1-R | TGGCGTCTTCCATGGTTCTCCACTTGCTATTACTTTAA | pGreenII0800-Pro_LTP1_ construction |
| 0800-LTP2-F | CAGCCCGGGGGATCCATGTAACACGATGTAAATC | pGreenII0800-Pro_LTP2_ construction |
| 0800-LTP2-R | TGGCGTCTTCCATGGTGATTAAACTTGTATTTT | pGreenII0800-Pro_LTP2_ construction |

The restriction sites used in the vector construction were underlined.

**Sequences of the promoters that used for activity evaluation**

**35S promter from pHB (531 bp):**

CATGGAGTCAAAGATTCAAATAGAGGACCTAACAGAACTCGCCGTAAAGACTGGCGAACAGTTCATACAGAGTCTCTTACGACTCAATGACAAGAAGAAAATCTTCGTCAACATGGTGGAGCACGACACACTTGTCTACTCCAAAAATATCAAAGATACAGTCTCAGAAGACCAAAGGGCAATTGAGACTTTTCAACAAAGGGTAATATCCGGAAACCTCCTCGGATTCCATTGCCCAGCTATCTGTCACTTTATTGTGAAGATAGTGGAAAAGGAAGGTGGCTCCTACAAATGCCATCATTGCGATAAAGGAAAGGCCATCGTTGAAGATGCCTCTGCCGACAGTGGTCCCAAAGATGGACCCCCACCCACGAGGAGCATCGTGGAAAAAGAAGACGTTCCAACCACGTCTTCAAAGCAAGTGGATTGATGTGATATCTCCACTGACGTAAGGGATGACGCACAATCCCACTATCCTTCGCAAGACCCTTCCTCTATATAAGGAAGTTCATTTCATTTGGAGAGAACACG

**2×35S promter from pHB (1307 bp):**

GAATTCGCCCGGGGATCTCCTTTGCCCCAGAGATCACAATGGACGACTTCCTATATCTCTACGATCTAGTCAGGAAGTTCGACGGAGAAGGTGACGATACCATGTTCACCACTGATAATGAGAAGATTAGCCTTTTCAATTTCAGAAAGAATCCTAACCCACAGATGGTTAGAGACGCTTACGCAGCAGGTCTCATCAAGACGATCTACCCGAGCAATAATCTCCAGGAGATCAAATACCTTCCCAAGAAGGTTAAAGATGCAGTCAAAAGATTCAGGACTAACTGCATCAAGAACACAGAGAAAGATATATTTCTCAAGATCAGAAGTACTATTCCAGTATGGACGATTCAAGGCTTGCTTCACAAACCAAGGCAAGTAATAGAGATTGGAGTCTCTAAAAAGGTAGTTCCCACTGAATCAAAGGCCATGGAGTCAAAGATTCAAATAGAGGACCTAACAGAACTCGCCGTAAAGACTGGCGAACAGTTCATACAGAGTCTCTTACGACTCAATGACAAGAAGAAAATCTTCGTCAACATGGTGGAGCACGACACGCTTGTCTACCTCCAAAAATATCAAAGATACAGTCTCAGAAGACCAAAGGGAATTGAGACTTTTCAACAAAGGGTAATATCCGGAAACCTCCTCGGATTCCATTGCCCAGCTATCTGTCACTTTATTGTGAAGATAGTGGAAAAGGAAGGTGGCTCCTACAAATGCCATCATTGCGATAAAGGAAAGGCCATCGTTGAAGATGCCTCTGCCGACAGTGGTCCCAAAGATGGACCCCCACCCACGAGGAGCATCGTGGAAAAAGAAGACGTTCCAACCACGTCTTCAAAGCAAGTGGATTGATGTGATAACATGGTGGAGCACGACACGCTTGTCTACCTCCAAAAATATCAAAGATACAGTCTCAGAAGACCAAAGGGAATTGAGACTTTTCAACAAAGGGTAATATCCGGAAACCTCCTCGGATTCCATTGCCCAGCTATCTGTCACTTTATTGTGAAGATAGTGGAAAAGGAAGGTGGCTCCTACAAATGCCATCATTGCGATAAAGGAAAGGCCATCGTTGAAGATGCCTCTGCCGACAGTGGTCCCAAAGATGGACCCCCACCCACGAGGAGCATCGTGGAAAAAGAAGACGTTCCAACCACGTCTTCAAAGCAAGTGGATTGATGTGATATCTCCACTGACGTAAGGGATGACGCACAATCCCACTATCCTTCGCAAGACCCTTCCTCTATATAAGGAAGTTCATTTCATTTGGAGAGGACACGCTGAAATCACCAGTCTCTCTCT

**g35S promter from GoldenBraid 2.0 Kit (1032 bp):**

GGAGACTAGAGCCAAGCTGATCTCCTTTGCCCCGGAGATCACCATGGACGACTTTCTCTATCTCTACGATCTAGGAAGAAAGTTCGACGGAGAAGGTGACGATACCATGTTCACCACCGATAATGAGAAGATTAGCCTCTTCAATTTCAGAAAGAATGCTGACCCACAGATGGTTAGAGAGGCCTACGCGGCAGGTCTGATCAAGACGATCTACCCGAGTAATAATCTCCAGGAGATCAAATACCTTCCCAAGAAGGTTAAAGATGCAGTCAAAAGATTCAGGACTAACTGCATCAAGAACACAGAGAAAGATATATTTCTCAAGATCAGAAGTACTATTCCAGTATGGACGATTCAAGGCTTGCTTCATAAACCAAGGCAAGTAATAGAGATTGGAGTCTCTAAGAAAGTAGTTCCTACTGAATCAAAGGCCATGGAGTCAAAAATTCAGATCGAGGATCTAACAGAACTCGCCGTGAAGACTGGCGAACAGTTCATACAGAGTCTTTTACGACTCAATGACAAGAAGAAAATCTTCGTCAACATGGTGGAGCACGACACTCTCGTCTACTCCAAGAATATCAAAGATACAGTCTCAGAAGACCAAAGGGCTATTGAGACTTTTCAACAAAGGGTAATATCGGGAAACCTCCTCGGATTCCATTGCCCAGCTATCTGTCACTTCATCAAAAGGACAGTAGAAAAGGAAGGTGGCACCTACAAATGCCATCATTGCGATAAAGGAAAGGCTATCGTTCAAGATGCCCCTGCCGACAGTGGTCCCAAAGATGGACCCCCACCCACGAGGAGCATCGTGGAAAAAGAAGACGTTCCAACCACGTCTTCAAAGCAAGTGGATTGATGTGATATCTCCACTGACGTAAGGGATGACGCACAATCCCACTATCCTTCGCAAGACCCTTCCTCTATATAAGGAAGTTCATTTCATTTGGAGAGGACTCCGGTATTTTTACAACAATTACCACAACAAAACAAACAACAAACAACATTACAATTTACTATTCTAGTCGA

**g2×35S promter from GoldenBraid 2.0 Kit (1333 bp):**

GGAGACTAGAGCCAGCTGATCTCCTTTGCCCCGGAGATCACCATGGACGACTTTCTCTATCTCTACGATCTAGGAAGAAAGTTCGACGGAGAAGGTGACGATACCATGTTCACCACCGATAATGAGAAGATTAGCCTCTTCAATTTCAGAAAGAATGCTGACCCACAGATGGTTAGAGAGGCCTACGCGGCAGGTCTGATCAAGACGATCTACCCGAGTAATAATCTCCAGGAGATCAAATACCTTCCCAAGAAGGTTAAAGATGCAGTCAAAAGATTCAGGACTAACTGCATCAAGAACACAGAGAAAGATATATTTCTCAAGATCAGAAGTACTATTCCAGTATGGACGATTCAAGGCTTGCTTCATAAACCAAGGCAAGTAATAGAGATTGGAGTCTCTAAGAAAGTAGTTCCTACTGAATCAAAGGCCATGGAGTCAAAAATTCAGATCGAGGATCTAACAGAACTCGCCGTGAAGACTGGCGAACAGTTCATACAGAGTCTTTTACGACTCAATGACAAGAAGAAAATCTTCGTCAACATGGTGGAGCACGACACTCTCGTCTACTCCAAGAATATCAAAGATACAGTCTCAGAAGACCAAAGGGCTATTGAGACTTTTCAACAAAGGGTAATATCGGGAAACCTCCTCGGATTCCATTGCCCAGCTATCTGTCACTTCATCAAAAGGACAGTAGAAAAGGAAGGTGGCACCTACAAATGCCATCATTGCGATAAAGGAAAGGCTATCGTTCAAGATGCCCCTGCCGACAGTGGTCCCAAAGATGGACCCCCACCCACGAGGAGCATCGTGGAAAAAGAAGACGTTCCAACCACGTCTTCAAAGCAAGTGGATTGATGTGATATCTCCACTGACGTAAGGGATGACGCACAATCCCACTATCCTTCGCAATGAGACTTTTCAACAAAGGGTAATATCGGGAAACCTCCTCGGATTCCATTGCCCAGCTATCTGTCACTTCATCAAAAGGACAGTAGAAAAGGAAGGTGGCACCTACAAATGCCATCATTGCGATAAAGGAAAGGCTATCGTTCAAGATGCCCCTGCCGACAGTGGTCCCAAAGATGGACCCCCACCCACGAGGAGCATCGTGGAAAAAGAAGACGTTCCAACCACGTCTTCAAAGCAAGTGGATTGATGTGATATCTCCACTGACGTAAGGGATGACGCACAATCCCACTATCCTTCGCAAGACCCTTCCTCTATATAAGGAAGTTCATTTCATTTGGAGAGGACTCCGGTATTTTTACAACAATTACCACAACAAAACAAACAACAAACAACATTACAATTTACTATTCTAGTCGA

***Arabidopsis thaliana UBQ10* (*AtUBQ10*) promoter from GoldenBraid 2.0 Kit (636 bp):**

GTCGACGAGTCAGTAATAAACGGCGTCAAAGTGGTTGCAGCCGGCACACACGAGTCGTGTTTATCAACTCAAAGCACAAATACTTTTCCTCAACCTAAAAATAAGGCAATTAGCCAAAAACAACTTTGCGTGTAAACAACGCTCAATACACGTGTCATTTTATTATTAGCTATTGCTTCACCGCCTTAGCTTTCTCGTGACCTAGTCGTCCTCGTCTTTTCTTCTTCTTCTTCTATAAAACAATACCCAAAGAGCTCTTCTTCTTCACAATTCAGATTTCAATTTCTCAAAATCTTAAAAACTTTCTCTCAATTCTCTCTACCGTGATCAAGGTAAATTTCTGTGTTCCTTATTCTCTCAAAATCTTCGATTTTGTTTTCGTTCGATCCCAATTTCGTATATGTTCTTTGGTTTAGATTCTGTTAATCTTAGATCGAAGACGATTTTCTGGGTTTGATCGTTAGATATCATCTTAATTCTCGATTAGGGTTTCATAGATATCATCCGATTTGTTCAAATAATTTGAGTTTTGTCGAATAATTACTCTTCGATTTGTGATTTCTATCTAGATCTGGTGTTAGTTTCTAGTTTGTGCGATCGAATTTGTCGATTAATCTGAGTTTTTCTGATTAACAG

***A. annua polyubiquitin-b* (*AaUBQb*) promoter (885 bp):**

GTTTGTTTTGGCTTATTTACCTGGCTTACGGCGTATTTGGTTTTGGGCTTATGTTTCATAAGTTTACTTTTTGTGGTTTATTTTAAAATTTTCCGGGTCATATAAGTTTAGTATGGCATAATAAGGTTATATAAGCTGAAAAGTTTCAAAATACGCCATAAGCCGGCTATATAAGCGAAACAAACACCCCAATCGATCAAGGCTTAATAAGTTTGAAAACATGTTTAAATAGTTATAAGTTGGAGCTTTATAGGTCTAAAAAGCTGTGTTGGTCGTAATTGGTTGGAGGAGCTATAGAGGTTCAAATACTAATGCTTAATAAGATGTCGGTTATGGTGTAACTAGCTTGATGATTTATGTCTTGAGGTCCAAGTTCTGCTTATGTATAGCTTTGTTATTGGTTGGAGCAAATAATGGGTAATAAGACGTGTAATTGTGTTCATGTAGTTACTAGTGTATGCTTAATTAGATATCGGGTTGTGTACCTGTTTTTGGTTTCTTTGATCTGCAATGAACTCCGAATTATCGTAATCAAGGGACTTGTATGGGATGCAATAATTAAAAACTAGTCTTTCAATACTAGAGTTGGCATTAGTCTAGGATTAGGTGATGAGTCGCACGTCTTTTATCAGGCGATATTATTGTTATAATTGATTTACGAGCTCTGATATATTTGCTAAGATATCTTGACAGTTTGCTGTATTATATTTTGTGGTTTTTGGGTATTTCAGTCTCTTGAGGGATTTTCGGCTTGCGATTGTAGTATACTAAATCTTGAACACTAGTTTGTTGCTTAGCTGATCCGATATGATATAACTGTTGCTGTTCTGTGGTATAATTTGTTGCTAAGGTTCTTAATGTGTTTTGATTTTTTATTTTGTGCAGATG
